# Pulse consumption of Australian adolescents: characteristics and consumption patterns in a national survey

**DOI:** 10.1017/S0007114526106308

**Published:** 2026-05-14

**Authors:** Adeline R. Lanham, Laura E. Marchese, Katherine M. Livingstone, Jessica R. Bogard, Jolieke C. van der Pols

**Affiliations:** 1 Faculty of Health, School of Exercise and Nutrition Sciences, Queensland University of Technology (QUT), Kelvin Grove, QLD, Australia; 2 Centre for Agriculture and the Bioeconomy, Queensland University of Technology (QUT), Brisbane, QLD, Australia; 3 Centre for the Environment and Society, Queensland University of Technology (QUT), Brisbane, QLD, Australia; 4 Institute for Physical Activity and Nutrition (IPAN), School of Exercise and Nutrition Sciences, Deakin University, Geelong, VIC, Australia; 5 Agriculture and Food, CSIRO, St Lucia, QLD, Australia

**Keywords:** Dietary pulses, Adolescent nutrition, Sustainable diets, Australia, Demographics

## Abstract

Increased consumption of pulses can support healthy and sustainable diets; however, consumption of pulses in Western populations is low. Adolescents are an often overlooked yet important target group as they develop attitudes and behaviours that influence food choices into adulthood. To understand patterns of consumption, this study aimed to analyse characteristics and consumption patterns of Australian adolescents who consume pulses. Secondary analysis of the Australian National Nutrition and Physical Activity Survey data from 2011–2012 was carried out to identify adolescent (12–17 years, *n* 1007, nationally representative of *n* 101 130) pulse consumers, compare their nutritional and demographic characteristics with non-consumers and describe frequency, types and amounts of pulses consumed. Consumption of pulses amongst adolescents is low, with only 6 % of adolescents (48 % males) reporting consumption of pulses. Pulse consumption was associated with healthier weight and diet characteristics. After adjusting for age, sex and socio-economic index, overweight or obese adolescents were less likely to consume pulses than adolescents of a healthy weight or underweight (OR = 0·82; 95 % CI 0·69, 0·99; *P* = 0·043). Adolescent pulse consumers reported consuming more vegetables, dietary fibre and Fe and less discretionary foods, saturated fat and added sugars than non-consumers. Baked beans were the most commonly consumed type of pulses, followed by pulses as an ingredient in a vegetarian meal such as dahl. Future strategies are recommended to promote the consumption of pulses amongst adolescents due to the low consumption level, with consideration of familiar and appealing dishes to support adolescents in achieving healthier and sustainable diets.

There is a global need to increase the sustainability and resilience of food systems, alongside the promotion of healthy and nutritious diets, to address the environmental impacts of agriculture and food production^(1,2)^. Concurrently, a malnutrition epidemic has contributed to 25 % of children and adolescents and 67 % of adults in Australia being overweight or obese^(3)^. Legumes, and particularly pulses, have been identified for their important role in providing environmentally sustainable nutrition^(4,5)^. Pulses are dried leguminous beans and peas, such as chickpeas, lentils and kidney beans, excluding legumes harvested for their oil content (peanuts and soybeans)^(6)^. Pulses provide a good source of protein, complex carbohydrates, fibre and micronutrients such as potassium, folate, non-haem Fe and Zn^(7)^. With this nutritional profile, pulses are classified in both the ‘vegetable’ and ‘lean meat and alternatives’ food groups in the Australian Dietary Guidelines^(8)^. Consumption of pulses is associated with a reduced risk of diet-related chronic health conditions such as obesity, diabetes, CVD and some cancers^(7)^. Additionally, pulses provide environmental benefits as they improve soil content through their nitrogen fixation properties, reducing pests, diseases and the need for fertilisers^(6)^. Pulses are associated with very low greenhouse gas production compared to the production of animal-sourced protein-rich foods^(1,6)^.

Despite their nutritional and environmental benefits, Australian data suggest that pulse consumption is well below national and international guidelines. On average, Australian adults consume 50 g of cooked pulses per week (∼1 tablespoon/d)^(9)^, which is 25 % of the intake recommended in the Australian Dietary Guidelines^(8)^ and 15 % of the amount recommended by the EAT-Lancet recommendations^(4)^. More detailed reports of pulse consumption patterns and the factors associated with pulse consumption across different age groups in Australia are lacking.

Adolescents are an important target group for efforts to improve nutritional and environmental outcomes of dietary habits. Adolescents (aged 12–17 years, aligning with high school years) in Australia are the least likely age group to meet the recommended intake of the ‘vegetable’ or ‘lean meat and alternatives’ food groups and source more than 40 % of their energy intake from discretionary foods including fast food, takeaway, soft drinks and baked goods^(9)^. During adolescence and the high school years, individuals develop knowledge, attitudes and skills relating to food choices, influencing food-related behaviour into adulthood^(10)^.

Increased healthiness and sustainability of diets consumed by adolescents can have long-lasting impacts during the remainder of their life course^(11)^. Interventions are needed to support such changes, which will require improved understanding of Australian adolescents’ consumption patterns of pulses and the demographic and nutritional characteristics of adolescent pulse consumers and non-consumers. Therefore, the aim of this study was to examine the nutritional and demographic characteristics of Australian adolescent pulse consumers and report on the patterns of pulse consumption, using the most recent national dietary intake data.

## Methods

This study was completed in compliance with the Strengthening the Reporting of Observational Studies in Epidemiology-Nutritional Epidemiology checklist^(12)^.

### Study sample

Data collected as part of the Australian Health Survey (2011–2013), including from the National Nutrition and Physical Activity Survey (NNPAS) and National Health Measures Survey, were analysed for this study using expanded confidentialised unit record files (CURF) accessed via the Australian Bureau of Statistics^(13)^. Details of the methods of the NNPAS and National Health Measures Survey can be found elsewhere^(14)^. In brief, detailed information was collected from one adult and up to one child across 9500 households in Australia, including dietary data (collected through one or two 24-h recalls) and demographic and anthropometric data for each participant (*n* 12 153) through the NNPAS, as well as biochemical indicators (serum) (*n* 11 000) through the NHMS. Households were sampled at random, and weights for each individual were calculated to infer representation at a national level. For the purpose of this study, demographical data from all adolescent survey participants (12–17 years) only were included (*n* 1007). Dietary intake data were collected by a trained interviewer with an adult from the household (adolescent involvement was encouraged, however optional), via face-to-face interview for the first recall (98 % adult response rate, 100 % adolescent response rate (*n* 1007)) and via telephone for the second recall (64 % adult response rate; 60 % adolescent response rate (*n* 608)). The food group and nutrient intake of each reported food consumed (including composite foods) was derived from the Australian Health Survey – Australian Dietary Guidelines database (2017) and Australian Food, Supplement and Nutrient 2011–2013 databases^(15,16)^, as reported in the expanded CURF^(13)^. As the expanded CURF data reported disaggregated food group intake for all food items based on the direct food group classification or recipes according to the Australian Health Survey – Australian Dietary Guidelines and Australian Food, Supplement and Nutrient 2011–2013 databases, pulse intake was reported as stated in the expanded CURF. Dietary intake data were assessed for reporting accuracy (see Nutritional Analysis below) and analysed for *n* 682 participants only. A figure outlining participant inclusion for each analysis can be found in the online Supplementary Material.

### Pulse consumption

Pulse consumers were identified as those reporting consumption of a pulse food item in either one or both 24-h recalls. Pulse-containing food items were analysed from the 1615 24-h recalls collected from 1007 adolescent participants, which included a total of 22 988 recorded food items. Pulse-containing foods were identified from the data as food items with Australian Food, Supplement and Nutrient 2011–2013 food codes containing ‘legume’ or ‘pulses’ in the food category (5 digit code, e.g. 25 101 ‘Mature legumes and pulses’) or food item (8 digit code, e.g. 25201005 ‘Baked beans, canned in tomato sauce, regular’) label, excluding those with tofu or soy beans^(16)^ (*n* 67). This included composite dishes (i.e. foods containing a mixture of meat and pulses).

### Data analysis

#### Study population and demographic analysis

Analysis was undertaken to describe the demographic and nutritional characteristics of pulse consumers and non-consumers and consumption patterns of pulses by Australian adolescents using Stata statistical software version 17.0^(17)^. Data and all analyses were weighted using replicate weight variables provided by the Australian Bureau of Statistics at the person level to adjust for the complex sampling design and ensure that data were representative of the larger Australian population^(13,18)^. Demographic data and dietary preferences/restrictions were analysed for all adolescent participants and separately for adolescent pulse consumers and non-consumers. This included age (in years), sex (male or female) country of birth (as Australia, main English-speaking countries or other countries), remoteness of residence (assessed with the Australian Statistical Geography Standard and categorised as major city, inner regional area or other (outer regional, remote and very remote areas))^(19)^, self-reported proficiency of English and socio-economic status (assessed with the Socio-Economic Index of Disadvantage for Areas quintiles (index combines an areas data for income, education, employment, occupation, housing and family structure))^(20)^. BMI categories were classified based on sex and age in half-years^(21,22)^. Dietary restrictions for cultural, religious or ethical reasons only were reported, to reflect participants’ dietary preferences and associated values rather than medical dietary requirements. Biochemical nutritional markers were also analysed for adolescent participants with available data; however, due to a small sample size (*n* 16 with biochemical data) and the associated low validity, the results have not been reported.

#### Nutritional analysis

For the purpose of quantitative nutritional analysis, participants were excluded if identified as an under- or over-reporter using a modified Goldberg cut-off ratio (energy intake: BMR), adjusted for each participant’s sex and physical activity level^(23)^. Physical activity levels were derived from the number of days each week participants reported participating in physical activity for greater than 1 h (0–2 d = light, 3–5 d = moderate, > 5 d = high)^(24)^. Using this method, 682 participants (67·7 %, population size = 1 174 028) were identified as likely accurate reporters for inclusion in the nutritional analysis. Overall, 15·1 % of respondents were identified as under-reporters, and 0·5 % were identified as over-reporters, and data to calculate reporting accuracy (weight or physical activity level) were missing for 16·7 % of participants; therefore, nutritional data for these participants were not analysed. There was no statistically significant difference in reporting bias (under- or over- reporting) between adolescent pulse consumers and non-consumers (*P* = 0·31).

Mean nutritional intake was calculated for each participant with available data from both days of the 24-h recalls. Weighted mean daily intake (with standard error) of each food group (wholegrains and cereals, fruits, vegetables, lean meats and alternatives, dairy products and alternatives, as well as discretionary foods) and the proportion of the population meeting recommended food group intake were reported overall and for both pulse consumers and non-consumers, with reference to the current Australian Dietary Guidelines, appropriate to each participant’s age and sex^(8)^. ‘Discretionary foods’, as defined by the Australian Dietary Guidelines and previously identified from the dataset^(8,15)^, were reported separately to the five food groups. Due to the nutrient profile and classification of pulses within both the vegetable and meat and alternative food groups of the Australian Dietary Guidelines^(8)^, pulses contributed to both food groups in this analysis^(15)^. Pulse consumption was also analysed separately and reported according to a 150 g serve size^(8)^.

Weighted mean dietary intakes of key nutrients relevant to the Australian adolescent population (energy, protein, dietary fibre, saturated fat, added sugar (as sugars added during processing or preparation or by the manufacturer or consumer^(16)^), Fe, Zn, vitamin B_12_, folate and Na) and proportion of the population meeting recommended nutrient intakes were reported overall and for both pulse consumers and non-consumers. Nutrient intake from supplements was not included in this analysis. Recommended nutrient intakes were in reference to the National Health and Medical Research Council’s Acceptable Macronutrient Distribution Ranges (protein, saturated fat and added sugar), micronutrient upper limit (for Na) and estimated average requirement (all other micronutrients), relevant for each individual participant’s age and sex^(25)^.

#### Statistical analysis

Using the weighted data, the difference in demographic and nutritional characteristics between pulse consumers and non-consumers was analysed using *t* tests to compare means and *χ* ^2^ tests to compare differences in proportions between adolescent pulse and non-consumers. Multivariable logistic regression was undertaken to determine key demographic variables associated with pulse consumption. Three models were used to determine (a) if overweight/obesity was associated with pulse consumption, independent of socio-economic index of residential area, age and sex; (b) whether country of birth was associated with consumption of pulses, independent of socio-economic index and regionality of residential area, household income, age and sex; or (c) the overall demographic characteristics associated with pulse consumption amongst adolescents. Statistical significance was indicated when *P* < 0·05.

#### Pulse consumption

Finally, pulse consumption patterns were analysed by the frequency and quantity of consumption of pulse-containing foods. Consumed pulses were grouped by food item type derived from the Australian Food, Supplement and Nutrient 2011–2013 classification system (e.g. canned (commercially sterile), legume-based dips, mixed dishes, etc.)^(16)^ and mealtime of consumption (as assigned from the 24-h recall data). Quantities of pulses were analysed as the mean number of grams of pulses consumed per person per eating occasion (a single incidence of consumption during any of the mealtimes). For the purpose of analysing pulse consumption, reports of pulse consumption for all participants were included (i.e. data from under- and over-reporters and those with missing weight or physical activity levels were included).

### Ethical approval

This analysis used the 2011–2012 NNPAS data, accessed with permission through the Australian Bureau of Statistics/Universities Australia Agreement. Ethical approval was not required to use the Expanded CURF, as per the Census and Statistics Act 1905^(13)^.

## Results

### Study population and demographic analysis

Demographic characteristics of participants are reported in Table 1. A total of 1007 adolescents (population size = 1 684 351; 51 % males) were included in this study, with an average age of 14·3 years. The majority of adolescents were born in Australia (89 %), with a portion born overseas from either predominantly English-speaking countries (UK, USA, Ireland, New Zealand, Canada, South Africa) (6 %) or predominantly non-English-speaking countries (5 %). All participants spoke mainly English at home (96 %) or spoke English very well or well (4 %). The majority of participants resided in major cities (66 %) or inner regional areas (23 %), with fewer from other regional or rural areas (11 %). Of those that reported body weight and height (84 %), most (68 %) participants reported a healthy BMI, 25 % were overweight or obese and 5 % were underweight. Regarding dietary preferences, a majority (95 %) of adolescents did not restrict their diet due to cultural, religious or ethical reasons (not including restrictions due to allergy/intolerance); however, the most common foods avoided for these reasons were pork (4 %), all meat (vegetarian) (1 %), poultry (1 %) and beef (1 %), with only 0·3 % avoiding all animal products (vegan).

**Table 1.** Participant characteristics of all adolescent respondents and adolescent pulse/non-consumers using weighted data from the Australian Health Survey (2011–2013) Table 1 long description.

	All adolescent respondents (*n* 1007, population size 1 684 351)			Adolescent pulse consumers (*n* 64, population size 101 130)			Adolescent non-consumers (*n* 943, population size = 1 583 221)			
	%	Mean	se	%	Mean	se	%	Mean	se	*P*
Age (mean (se), years)		14·3	0·08		14·7	0·18		14·3	0·08	**0·04**
Male (%)	51			48			51			0·69
Country of birth (%)										0·16
Australia	89			84			89			
Other major English-speaking countries*	6			5			6			
Other countries	5			11			5			
Regionality of residence^†^ (%)										0·95
Major city	66			68			66			
Inner regional area	23			23			23			
Other	11			9			11			
SEIFA^‡^ (%)										0·94
Lowest 20 %	16			13			16			
Second quintile	19			20			19			
Third quintile	23			26			22			
Fourth quintile	19			21			19			
Highest 20 %	23			19			23			
Proficiency of English (%)										0·69
Mainly speaks English at home	96			95			96			
Well/very well	4			5			4			
Not well/not at all	0			0			0			
Not known/does not speak	0			0			0			
Equivalised income of household^§^ (%)										**0·0031**
First decile	10			9			10			
Second decile	5			5			5			
Third decile	8			6			9			
Fourth decile	9			3			9			
Fifth decile	12			30			11			
Sixth decile	12			5			13			
Seventh decile	9			16			8			
Eighth decile	8			12			8			
Ninth decile	6			1			6			
Tenth decile	5			9			5			
Not reported	15			4			14			
BMI category^||^ (%)										0·17
Underweight	6			9			6			
Healthy weight range	68			81			67			
Overweight or obese	25			10			26			
Dietary avoidances (non-allergy) (%)										
All animal products (vegan)	0·3			1·1			0·2			0·24
All meat (vegetarian)	1·1			3·4			1·0			**0·05**
Pork	4·0			7·6			3·7			0·21
Beef	0·8			5·7			0·5			**0·01**
Poultry	0·9			5·8			0·6			**0·01**
No avoidances	94·6			89·3			95·0			0·10
Physical activity level^¶^ (%)										**0·0359**
Low	40			37			41			
Medium	30			55			39			
High	20			8			21			
Unknown	< 1			1			< 1			

SEIFA, Socio-Economic Indexes for Areas.

*P*-values < 0·05 (in bold) indicate statistically significant difference between pulse consumers and non-consumers in the category below using Pearson’s *χ* ^2^ test.

*Other major English-speaking countries include Canada, the Republic of Ireland, New Zealand, South Africa, the UK and the USA.

^†^Regionality according to the Australian Statistical Geography Standard^(19)^.

^‡^SEIFA ranks areas according to relative socio-economic disadvantage, quintiles.

^§^Equivalised by household size, deciles.

^||^BMI categories relative to sex and age in half-years, percentage of those with reported weight.

^¶^Number of days did physical activity for at least 1 h in 7 d prior to day 1 survey (low = 0–2 d, medium 3–5 d, high = 6–7 d).

### Pulse consumers demographic analysis

Of the 1007 adolescent participants (population size = 1 684 351), 64 (6 %) participants (48 % males) reported consuming pulses (nationally representative of 101 130 Australian adolescents). Pulse consumers were reported to be slightly older (14·7 years *v*. 14·3 years, *P* = 0·04) than non-consumers; however, there was no significant difference in sex (*P* = 0·69). Pulse consumers were more likely than non-consumers to avoid some kind of animal containing foods due to cultural, religious or other reasons (10·7 % *v*. 5 %, *P* = 0·10), with significant difference in dietary avoidance of beef (5·7 % *v*. 0·5 %, *P* < 0·01), poultry (5·8 % *v*. 0·6 %, *P* < 0·01) and all meat (i.e. vegetarian) (3·4 % *v*. 1·0 %, *P* = 0·05). When adjusted for socio-economic index, area of residency, household income, age and sex, pulse consumption was not significantly associated with country of birth (OR = 1·21; 95 % CI 0·40, 3·65; *P* = 0·74). Of those with recorded weights (80 % for pulse consumers, 84 % for non-consumers), pulse-consuming adolescents were also more likely to have a healthy BMI (81 % *v*. 67 %) and less likely to be overweight or obese (10 % *v*. 26 %); however, these differences were not statistically significant (*P* = 0·14). When adjusted for age, sex and socio-economic index, overweight or obese adolescents were less likely to consume pulses than adolescents of a healthy weight or underweight (OR = 0·82; 95 % CI 0·69, 0·99; *P* = 0·043). Furthermore, with all predictor variables included in the model, BMI was the only variable that was significantly associated with consumption of pulses, with the results indicating that adolescents who are overweight or obese are less likely than those of a healthy weight or underweight to consume pulses (OR = 0·78; 95 % CI 0·61, 1·00; *P* = 0·05).

### Nutritional analysis – adolescents overall

From the 682 participants identified as likely accurate reporters (population size = 1 174 028), the majority of adolescents did not meet the recommended number of serves from any of the food groups outlined in the Australian Dietary Guidelines (see Table 2). In particular, there was low daily consumption of vegetables (mean = 2·1 serves (se = 0·08), with 7 % meeting recommended intake (se = 0·01)), dairy products and alternatives (mean = 1·7 serves (se = 0·09), with 12 % meeting recommended intake (se = 0·02)) and lean meats and alternatives (mean = 1·8 serves (se = 0·07), with 25 % meeting recommended intake (se = 0·02)). There was moderate to high intake of discretionary and/or processed foods amongst Australian adolescents overall with an average consumption of 7·4 serves per d of discretionary foods (se = 0·20) (16 % meeting recommendation (se = 0·02)) and average daily added sugars consumption of 78 g/18·5 teaspoons (se = 2·71). The average reported percentage of daily energy intake from added sugars (13 % (se = 0·37)) also exceeded the recommendations of added sugars to contribute < 10 % of daily energy intake, with only 39 % of adolescents reporting a diet meeting these recommendations (se = 0·03). Recommended micronutrient intakes for each of Fe, Zn, vitamin B_12_ and folate were met by the majority (> 80 %) of adolescents; however, only 35 % (se = 0·02) of participants met the recommended intake of dietary fibre (mean = 22·8 g (se = 0·43); recommended = 22 g for females, 28 g for males), and only 34 % (se = 0·02) of reported diets were within the recommended upper limit for Na (mean = 2852 mg (se = 56·83); recommended upper limit = 2000 mg for 12–13 year olds, 2300 mg for > 13 year olds).

**Table 2.** Food and nutrient intake of all adolescents and adolescent pulse and non-consumers using weighted data from the Australian Health Survey (2011–2013)

Nutrient or food group	Recommended intake^*^	Daily intake	All respondents (*n* 682, population size = 1 174 028)		Pulse consumers (*n* 49, population size = 75 965)		Non-consumers (*n* 633, population size = 1 099 062)		*P*
Wholegrains and cereals	5 serves (12 and 13 yo females); 6 serves (12 and 13 yo males); 7 serves (14+yo)	Mean number of serves	5·1	se = 0·12	5·9	se = 0·55	5·0	se = 0·12	0·13
		% of population meeting recommendations	37	se = 0·03	46	se = 0·08	36	se = 0·03	0·29
Fruit	2 serves	Mean number of serves	1·6	se = 0·08	1·7	se = 0·15	1·6	se = 0·08	0·42
		% of population meeting recommendation	31	se = 0·02	40	se = 0·10	30	se = 0·02	0·27
Vegetables	5 serves (females); 5·5 serves (males)	Mean number of serves	2·1	se = 0·08	3·9	se = 0·36	1·9	se = 0·09	**< 0·001**
		% of population meeting recommendation	7	se = 0·01	19	se = 0·07	6	se = 0·01	**0·007**
Lean meats and alternatives	2·5 serves	Mean number of serves	1·8	se = 0·07	2·4	se = 0·32	1·8	se = 0·07	0·06
		% of population meeting recommendation	25	se = 0·02	38	se = 0·10	24	se = 0·03	0·13
Pulses	–	Mean number of serves (150 g)	0·05	se = 0·01	0·7	se = 0·16	< 0·1	se = 0·00	**< 0·001**
Dairy products and alternatives	3·5 serves	Mean number of serves	1·7	se = 0·09	1·5	se = 0·18	1·7	se = 0·09	0·30
		% of population meeting recommendation	12	se = 0·02	8	se = 0·05	12	se = 0·02	0·52
Discretionary	0–2·5 serves (females), 0–3 serves (12 and 13 yo males) 0–5 serves (14+yo males)	Mean number of serves	7·4	se = 0·20	5·7	se = 0·52	7·5	se = 0·21	**0·002**
		% of population meeting recommendation	16	se = 0·02	27	se = 0·09	16	se = 0·02	0·14
Energy		Mean intake (kJ)	10 021	se = 122·57	9580	se = 460·53	10 051	se = 131·77	0·35
Protein	15–25 % energy intake (AMDR)	Mean intake (g)	96	se = 1·70	95	se = 6·49	96	se = 1·84	0·89
		Mean intake (% of energy intake)	16	se = 0·20	16	se = 0·59	16	se = 0·20	0·66
		% of population meeting recommendation	54	se = 0·03	62	se = 0·09	54	se = 0·03	0·33
Saturated fat	< 10 % of energy intake (AMDR)	Mean intake (g)	37	se = 0·80	32	se = 1·92	37	se = 0·84	**0·007**
		Mean intake (% of energy intake)	14	se = 0·20	13	se = 0·39	14	se = 0·22	**< 0·001**
		% of population meeting recommendation	13	se = 0·02	9	se = 0·05	13	se = 0·02	0·49
Dietary fibre	22 g (females); 28 g (males) (AI^‡^)	Mean intake (g)	22·8	se = 0·43	31·1	se = 2·07	22·3	se = 0·42	**< 0·001**
		% of population meeting recommendation	35	se = 0·02	75	se = 0·08	33	se = 0·02	**< 0·001**
Added sugars	< 10 % energy intake (AMDR)	Mean intake (g)	78	se = 2·71	55	se = 6·62	79	se = 2·98	**0·003**
		Mean intake (% of energy intake)	13	se = 0·37	10	se = 0·94	13	se = 0·41	**0·002**
		% of population meeting recommendation	39	se = 0·03	71	se = 0·07	37	se = 0·03	**< 0·001**
Na	2000 mg (12 and 13 yo); 2300 mg (14+yo) (UL)	Mean intake (mg)	2852	se = 56·83	2685	se = 212·86	2863	se = 60·95	0·44
		% of population meeting recommendation	34	se = 0·02	44	se = 0·10	33	se = 0·03	0·24
Fe	6 mg (12–13 yo); 8 mg (14 yo+) (EAR)	Mean intake (mg)	12·0	se = 0·26	14·8	se = 1·34	11·8	se = 0·26	**0·033**
		% of population meeting recommendation	83	se = 0·02	90	se = 0·05	83	se = 0·02	0·21
Zn	5 mg (12 and 13 yo); 6 mg (14+yo females); 11 mg (14+yo males) (EAR)	Mean intake (mg)	11·6	se = 0·28	12·8	se = 1·49	11·5	se = 0·30	0·42
		% of population meeting recommendation	85	se = 0·02	85	se = 0·07	85	se = 0·02	0·94
Vitamin B_12_	1·5 mg (12 and 13 yo); 2 mg (14+yo) (EAR)	Mean intake (µg)	4·8	se = 0·14	4·4	se = 0·41	4·8	se = 0·15	0·45
		% of population meeting recommendation	93	se = 0·01	87	se = 0·06	93	se = 0·01	0·22
Folate equivalent	250 µg (12 and 13 yo); 330 µg (14+yo) (EAR)	Mean intake (µg)	710	se = 16·86	805	se = 65·91	704	se = 16·89	0·13
		% of population meeting recommendation	94	se = 0·01	99	se = 0·01	93	se = 0·01	0·07

yo, years old; AMDR, Acceptable Macronutrient Distribution Range; AI, adequate intake; UL, upper level of intake; EAR, estimated average requirement.

*P*-values < 0·05 (in bold) indicate statistically significant difference between pulse consumers and non-consumers in the category below using logistic regression of weighted data.

Shading of cells according to the proportion of population meeting recommendations, in quintiles.

* Derived from national Nutrient Reference Values and the Australian Dietary Guidelines relative to sex and age in years (yo)^(8,25)^.

### Nutritional analysis – comparing adolescent pulse/non-consumers

**Figure 1.**
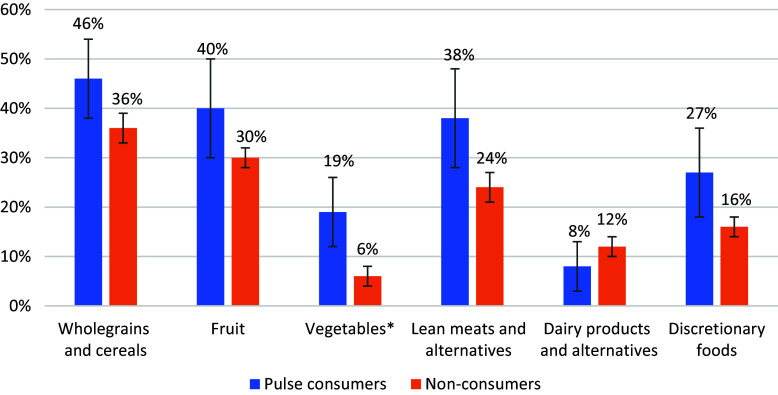
Australian adolescent pulse consumers and non-consumers meeting recommended serves for key food groups according to the Australian Dietary Guidelines^(8)^. Using survey weighted data from the Australian Health Survey 2011–2013, population size of pulse consumers = 101 130 and non-consumers = 1 583 221.* indicates statistically significantly different using Pearson’s *χ* ^2^ test.

The nutritional intake of adolescent pulse consumers differed from that of non-consumers (see Table 2). Pulse consumers consumed a greater number of serves of vegetables (3·9 (se = 0·36) *v*. 1·9 serves (se = 0·09), *P* < 0·001), with 19 % (se = 0·07) (*v*. 6 % (se = 0·01)) of pulse consumers meeting the recommended number of serves of vegetables (*P* = 0·0070) (see Figure 1). Pulse consumers also consumed more serves of lean meats and alternatives (2·4 (se = 0·32) *v*. 1·8 (se = 0·07) serves, *P* = 0·06), with 38 % (se = 0·10) meeting the recommended number of serves of meats and alternatives (*v*. 24 % (se = 0·03)) (*P* = 0·13). However, pulse consumers, on average, consumed less serves of dairy products and alternatives (1·5 (se = 0·18) *v*. 1·7 serves (se = 0·9), *P* = 0·30), with only 8 % (se = 0·05) of pulse consumers meeting the recommended number of serves of dairy products and alternatives (*v*. 12 % (se = 0·02). Pulse consumers consumed significantly less serves of discretionary foods than non-consumers (5·7 (se = 0·52) *v*. 7·5 (se = 0·21) serves, *P* = 0·002). Furthermore, the proportion of participants consuming an amount of discretionary foods within the recommendations for their age and sex was greater for pulse consumers than non-consumers (27 % (se = 0·09) *v*. 16 % (se = 0·02), *P* = 0·14); however, this difference was not statistically significant.

**Figure 2.**
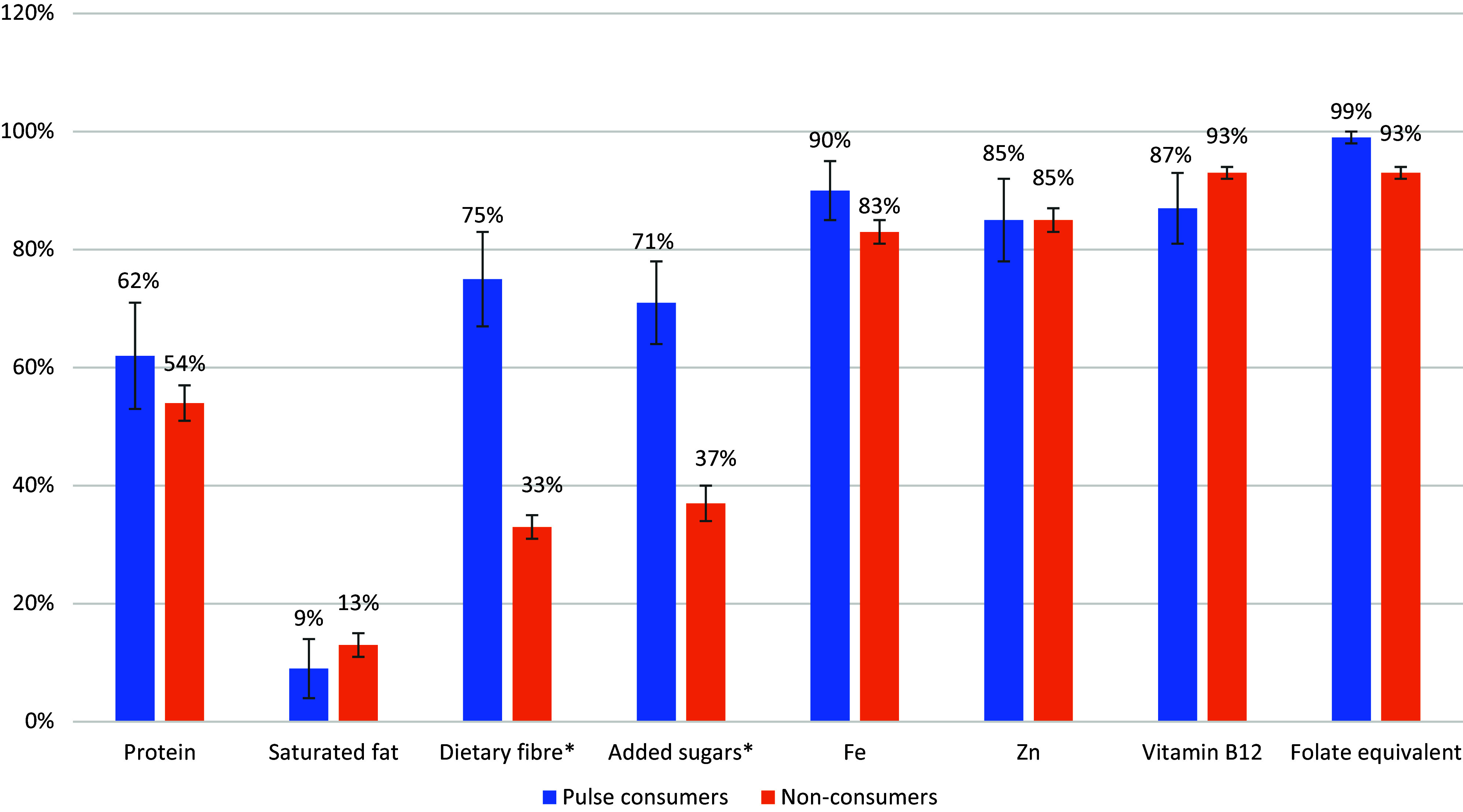
Australian adolescent pulse consumers and non-consumers meeting recommended dietary nutrient intake according to the 2017 Nutrient Reference Values^(25)^. Using survey weighted data from the Australian Health Survey 2011–2013, population size of pulse consumers = 101 130 and non-consumers = 1 583 221.* indicates statistically significantly different using Pearson’s *χ* ^2^ test.

Additionally, participants consuming pulses consumed significantly more dietary fibre (31 g (se = 2·07) *v*. 22 g (se = 0·42), *P* = 0·001) and less added sugars (55 g (se = 6·62) *v*. 79 g (se = 2·98), *P* = 0·003), with 75 % (se = 0·08) of pulse consumers meeting the recommended intake of dietary fibre (*v*. 33 % (se = 0·03), *P* < 0·001) and 71 % (se = 0·07) of pulse consumers below the recommended limit for added sugars (*v*. 37 % (se = 0·03), *P* < 0·001) (see Figure 2). Although not statistically significant, the mean Na intake was lower amongst pulse consumers (2685 mg (se = 212·86) *v*. 2863 mg (se = 60·95), *P* = 0·44), and a greater proportion of pulse consumers maintained a Na intake below the recommended upper limit (44 % (se = 0·10) *v*. 33 % (se = 0·03), *P* = 0·24) than non-consumers.

There was no statistically significant difference in the proportion of adolescents meeting the recommended intakes for Fe, Zn, vitamin B_12_ or folate. Amongst these micronutrients, the proportion of participants reporting intakes that met the recommended value was lowest for Fe (90 % (se = 0·03) for pulse consumers *v*. 83 % (se = 0·02) for non- consumers, *P* = 0·21), with the mean intake of Fe significantly greater amongst pulse consumers (14·8 mg (se = 1·34) for pulse consumers *v*. 11·8 mg (se = 0·26) for non-consumers, *P* = 0·03). Greater than 85 % of respondents (both pulse consumers and non-consumers) met the estimated average requirements for Zn and vitamin B_12_, whilst the proportion of respondents meeting recommended folate intake was high in both groups (99 % (se = 0·01) for pulse consumers *v*. 93 % (se = 0·01) for non-consumers, *P* = 0·07.

### Pulse consumption

**Figure 3.**
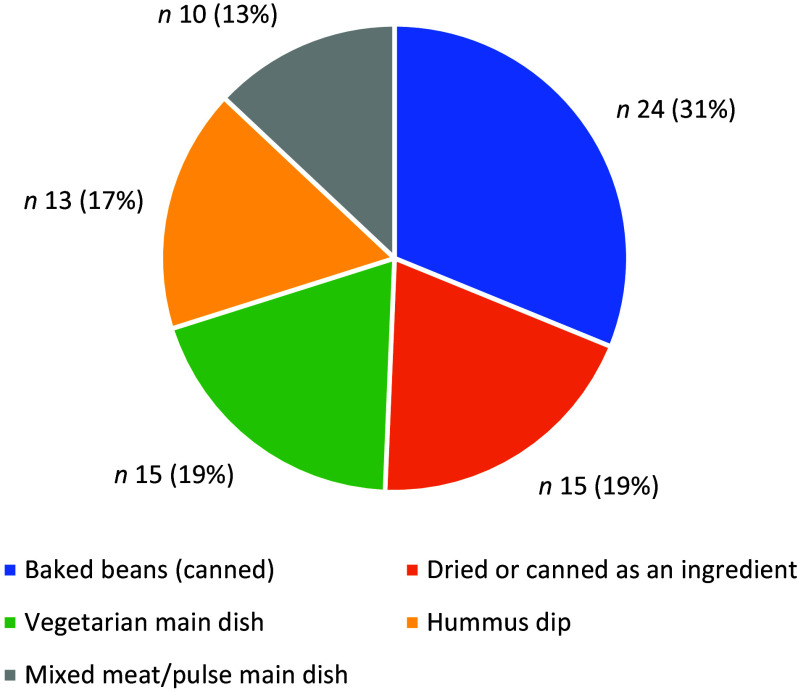
Food items consumed containing pulses (*n* 77).

From the sixty-four pulse consumers (including under- and over-reporters), there were seventy-seven (0·3 %) reports of consumption of pulse-containing food items, from twenty-nine different pulse food items. As displayed in Figure 3, the most commonly consumed pulse food item was baked beans (*n* 24, 31 %), followed by legumes as an individual ingredient (*n* 15, 19 %), in vegetarian main dishes (casserole, soup, curry, dahl, patty, salad) (*n* 15, 19 %), hummus dip (*n* 13, 17 %) or in mixed meat/pulse main dishes (e.g. casserole, soup, curry) (*n* 10, 13 %). When consumed as an ingredient, pulses were more commonly prepared from a dried product (*n* 12) than canned (*n* 3).

Per day, the mean number of serves of pulses consumed by pulse consumers was 0·7 serves (se = 0·16) (of a 150 g serve of ‘lean meats and alternatives’). The mean portion size of pulses consumed was 161 g, with the greatest mean portion size consumed as baked beans (223 g) or within a vegetarian main dish (183 g), followed by as an ingredient from a cooked dried or canned food (139 g) and mixed meat/pulse main dishes (129 g). Pulses consumed in dips were consumed in smaller amounts (a mean of 23 g).

The mealtime at which pulse-based food items were consumed varied depending on the type of food item. Overall, pulses were most commonly reported to be consumed at dinner (*n* 39, 51 %), followed by as a snack or mid-meal (*n* 14, 18 %), breakfast (*n* 12, 16 %), lunch (*n* 11, 14 %) or brunch (*n* 1, 1 %). Pulses used as an individual ingredient or as part of either a vegetarian or mixed meat/pulses main dish were most commonly consumed at dinner (*n* 27, 70 %), whereas baked beans were similarly consumed at dinner (*n* 11, 46 %) or breakfast (*n* 10, 42 %) or less often for lunch (*n* 3, 13 %). On the other hand, hummus was more commonly consumed as a snack or at mid-meals (*n* 9, 70 %) rather than with a main meal (*n* 4, 31 %).

## Discussion

This secondary analysis of the 2011–2012 NNPAS describes the nutritional and demographic characteristics of Australian adolescents (pulse consumers and non-consumers) and reports on adolescents’ consumption patterns of pulses. The results from this analysis identify that pulse consumption amongst adolescents is low and that the dietary intake of Australian adolescents across all food groups does not meet the 2013 Australian Dietary Guidelines^(8)^. Consumption of pulses in this age group is associated with reduced risk of overweight or obesity, as well as statistically significant increased consumption of vegetables and dietary fibre, and reduced intake of added sugars. Pulses are most commonly consumed by adolescents at main meal times, in the form of baked beans or as part of a main dish.

The low level of pulse consumption in this age group (6 %) identified that very few Australian adolescents meet the recommended intake of two serves per week^(8)^. The increased likelihood for pulse consumers to avoid some animal foods may be confounding for a cultural, religious and/or ethical norm in which pulses are used as a staple food or key protein source and consumed more regularly^(26)^. Amongst adults from Asian, African or South American backgrounds in which pulses feature commonly in traditional dishes, relatively frequent consumption of pulses has been observed in Australia and from global data^(26,27)^. In our analyses, there was no association between the participants’ country of birth and pulse consumption; however, cultural influences may still have been present if the parents or other carers, who may be primarily responsible for the provision of foods, originated from a cultural background in which pulses form a more common part of the cuisine^(28)^.

Our findings indicate a negative association between overweight/obesity and pulse consumption, which supports evidence of pulse consumption playing a role in the prevention of obesity^(29)^. As identified in this study, the mechanism for this may not be only due to the nutrient content of pulses themselves but also their culinary use and associated dietary patterns. Pulses are often consumed with fibre-rich, minimally processed foods including vegetables, which are associated with a healthy weight^(30,31)^.

The nutritional intake analysis of data from this national survey identifies concerns about the consumption of each of the five food groups outlined in the 2013 Australian Dietary Guidelines among Australian adolescents overall, which supports studies describing the intake from previous surveys or younger children^(32,33)^. In particular, the low intake of lean meats and alternatives and dairy products and alternatives, along with the low protein intake and high saturated fat intake, suggests that processed or lower quality meat and dairy products and alternatives are being consumed by adolescents. Likewise, although the amounts of wholegrains and cereals and fruit consumed by adolescents were closer to the recommended intake than other food groups, on average, the high added sugar, high Na and low dietary fibre intakes indicate that foods consumed within these food groups also may be sourced from processed foods. This also reflects the high consumption of discretionary foods in our study population.

The differences between pulse consumers and non-consumers in the number of serves consumed from the food groups suggest that pulses were consumed with other vegetables and in addition to other lean meats and alternatives (rather than as a substitute) by adolescents. The categorisation of pulses as both vegetables and lean meat and alternatives may have contributed to greater consumption of both these two food groups; however, the greater number of serves of vegetables consumed by pulse consumers cannot be accounted for by the contribution of pulses alone. This concurrent consumption of additional vegetables aligns with previous literature reporting commonly consumed pulse-containing foods to be mixed dishes with vegetables, with or without lean meat, and may be served with wholegrains such as Mexican bean dishes and mixed meat and pulse soups, curries and casseroles^(5,27)^. These dishes are typically minimally processed with limited added sugar and salt and may contribute to the lower intake of Na, added sugar and saturated fat, as well as higher intake of dietary fibre in adolescents who consumed pulses. Along with a lower intake of discretionary foods and lower energy intake, this may partly explain why there is a lower proportion of overweight or obese adolescents amongst pulse consumers.

Our analysis found that avoiding meat products is associated with pulse consumption; however, the total number of serves of lean meats and alternatives was greater amongst pulse consumers than non-consumers. It is known that those who restrict animal-sourced foods (such as vegetarians and vegans) have reduced intake of Fe, Zn and vitamin B_12_^(34)^. The lack of a significant difference in the proportion of adolescents meeting the recommended intakes for Fe, Zn and vitamin B_12_ suggests that pulse consumption does not increase the risk of inadequate dietary intake of these nutrients amongst adolescents. Indeed, our findings indicate that protein and Fe intake was higher amongst pulse consumers than non-consumers. However, this total Fe intake does not account for the reduced bioavailability of non-haem Fe from plant-based sources and is complex to estimate given the diverse dietary intakes, consumption patterns and unknown Fe status of consumers^(35)^. In this analysis, the Fe intake from animal sources is assumed to be similar between meat-eating pulse consumers and non-pulse consumers, as any pulses consumed appear to be consumed in addition to other serves of meat and alternatives. Therefore, any additional non-haem Fe intake from pulses would only increase the total Fe intake. However, this analysis was from cross-sectional data and cannot predict the impact on nutrient intake, resulting from the promotion of pulse consumption, in which the consumption of other foods (such as meat) may be reduced. Future intervention studies promoting pulse consumption that may result in reduced meat consumption should assess the intake and bioavailability of key nutrients such as Fe, Zn and vitamin B_12_ to reduce the risk of deficiency.

Amongst pulse consumers, the average quantity of pulses consumed was 105 g/d (1·4 serves of vegetables or 0·7 serves as a meat alternative). The 2013 Australian Dietary Guidelines recommend consuming two serves of legumes per week (as either a 75 g cooked (1/2 cup) serve as a vegetable or 150 g cooked (1 cup) serve as a meat alternative) for adolescents^(36)^. As the NNPAS dietary intake data were collected via 24-h recalls across 1–2 d, the frequency of pulse consumption across the week cannot be compared to the weekly recommendations. However, the reported intake of 0·7 serves of pulses per d (105 g) is lower than international recommendations, which suggest consumption of 150 g of cooked pulses per d (for a reference diet of approximately 10 MJ, similar to that reported by Australian adolescents) for optimal human and environmental health^(4)^. Thus, the quantity of pulses consumed by Australian adolescents is below recommendations; however, as discussed earlier, the prevalence of consumption is concerning, with only 6 % of adolescents reporting any consumption of pulses on either or both days of data collection.

This study did not assess the factors that explain the low prevalence of pulse consumption amongst Australian adolescents. Previous studies have identified barriers to pulse intake amongst adults including a lack of knowledge or skills of how to cook or incorporate pulses into meals, dislike of taste, unpleasant gastrointestinal symptoms (bloating, gas) and a perception that pulses are suitable only for people with vegetarian or vegan diet identities^(27,37)^. Barriers to pulse consumption specifically by adolescents have not been reported in the literature; however, an adolescent’s socio-economic and geographical context, their role in food preparation and the convenience and taste of foods are known to influence food choice and consumption more broadly^(10,38)^.

Aligning with previous literature from adults, Australian adolescents most commonly consumed pulse-containing food items at a main meal, with other ingredients commonly including vegetables (either in a vegetarian or mixed meat/pulse main dish or as baked beans)^(27)^. The variety of pulse-containing food items consumed also demonstrates the culinary versatility of pulses, which has previously been reported as a facilitator of their consumption amongst adults, along with health and nutritional benefits, low cost and convenience to prepare pulse dishes^(27,37,39)^. Promotion of familiar and commonly consumed pulse-containing foods, most commonly consumed such as soup, curry, dahl, patties, as well as baked beans and hummus products, may be more easily accepted by adolescents^(40)^.

There are direct implications from this analysis for both research and practice. Future studies may benefit from considering cultural background, socio-economic status and food literacy of adolescents and their families to further identify factors influencing consumption of pulses and therefore guide future strategies to promote pulse consumption. Additionally, understanding adolescents’ (and parents’, peers’ and schools’) role in the sourcing and preparation of their dietary intake may assist in identifying key stakeholders with which to engage for future pulse-promoting strategies. Importantly, future studies should identify the types and forms of pulses consumed by adolescents that reflect currently available products, and the nutritional and environmental impact of these pulse foods should also be considered prior to their promotion. Future interventions targeting the promotion of pulse consumption amongst adolescents may be more effective when making gradual introductions and inclusions of pulses in the diet, using foods already familiar to the target population^(41)^.

### Strengths and limitations

This study provides the first analysis of pulse consumption amongst Australian adolescents and the demographic and nutritional characteristics of adolescent pulse consumers. In using the NNPAS data, the most recent nationwide data were used, and the representativeness of demographic and nutritional data was increased by the use of the complex weights and exclusion of data from identified under- and over-reporters. However, a number of limitations impact the validity and applicability of this analysis.

The demographic and nutritional characteristics of participants from the 2011–2012 NNPAS were similar to those reported in more recent national data from 2014–15 and 2017^(42,43)^. The proportion of accurate reporters (18 % of those with reported weight +/– physical activity level) and mean energy intake were similar to those reported previously in the literature for Australian adolescents^(25,44)^. Due to inherent reporting bias and limitations of the survey methodology (see limitations below), the reported dietary intakes may not accurately report the quantity or full variety of types of foods. However, foods that are typically under-reported are those that are high in fat and sugar (i.e. discretionary foods)^(45)^, and therefore, the impact of reporting bias on the identification of pulse consumers and quantity of pulses consumed is expected to be limited.

The number of participants identified as pulse consumers was relatively small (*n* 64) despite the data sourced from the largest nationally representative survey available (*n* 1007, representative population size = 1 684 351). This may limit the strength of inferences regarding the demographical and nutritional characteristics of adolescent in particular when pulse consumers and non-consumers were compared. Dietary intake data from a nationally representative sample of Australian adolescents are needed.

As previously mentioned, the 24-h recall method for dietary intake assessment used in the NNPAS has limitations because these data may not be fully representative of typical consumption across weekdays and weekend days. Furthermore, in Australia, pulses are typically consumed episodically, not habitually, and therefore, consumption may not be best captured using the 24-h recall method. This may have led to some incorrect classification of adolescents as ‘non-consumers’. The extent of this potential misclassification is reduced by the inclusion of data from 2 d of 24-h recalls, where available. Future studies reporting on the prevalence and frequency of pulse consumption should use FFQ to estimate intake over longer time frames or, in populations where there is evidence of habitual consumption of pulses, use the multiple source method to assess usual intake from 24-h recalls. Additionally, the quantity of pulses consumed within these foods may be misrepresented in these data as ambiguous ‘legume’ containing foods (e.g. ‘soup with legumes’) were included as pulse-containing food items and may contain varying quantities of legumes (e.g. homemade pea and ham soup *v*. canned minestrone soup). The mode of data collection methods of the 24-h recall differed between the first and second days (face-to-face and telephone, respectively), with suggestions of mis- or underreporting from the second day due to a significantly lower mean energy intake^(46)^. Due to the overall low prevalence of pulse consumption, dietary intake data from both days were included in our analysis from participants identified as accurate reporters. Furthermore, as dietary data for each adolescent were collected from an adult in the household (with or without adolescent contribution), some foods, particularly those consumed outside the home, may not have been reported. This may have influenced the identification and quantification of pulse consumption from these data.

When undertaking the nutritional analysis, limitations of the referenced recommended intakes of nutrients should be acknowledged. The EAR was used for evaluating the inadequacy of Fe, Zn, B_12_ and folate intake amongst the Australian adolescent population^(25)^; however, this does not indicate inadequate micronutrient intake of individuals, which may vary depending on individual needs.

The data used were from a dataset over a decade old (at the time of analysis) and had some internal methodological limitations. The food landscape in Australia has changed over the past 10 years, particularly with an increase in the availability and consumption of plant-based foods, some of which are based on pulses (i.e. commercially available pre-prepared chickpea or lentil patties, vegetarian lasagnes, etc.)^(39,47–49)^. Consumption of many of these products was not indicated in the 2011–2013 Australian Health Survey, and it is expected that a cultural shift since the collection of these data may have led to increased consumption of processed pulse products^(50)^. However, more recent consumption data of these pulse foods amongst Australian adolescents is lacking – new national survey data will facilitate comparisons with the data presented in our study.

### Conclusions

This national survey indicates that consumption of pulses amongst Australian adolescents is low, both in terms of how many adolescents report consumption of pulses and in total intake amounts, which are below guidelines on average for those who reported consumption of pulses. Pulse consumers were more likely to avoid meat products; however, they maintained similar intake of Fe, Zn and vitamin B_12_ to non-consumers. Adolescent pulse consumers, on average, consumed more vegetables and dietary fibre and less saturated fat and added sugar, indicating dietary benefits beyond those associated with the consumption of the pulse foods alone. Future studies should focus on the best ways to encourage consumption of pulses amongst Australian adolescents to support healthy and sustainable dietary shifts for future generations.

## Supporting information

Lanham et al. supplementary material Lanham et al. supplementary material

## Supplementary material

For supplementary material/s referred to in this article, please visit https://doi.org/10.1017/S0007114526106308.

## Table 1 Long description

The table presents demographic characteristics of adolescent respondents, pulse consumers, and non-consumers. It includes data on age, gender, country of birth, region of residency, socioeconomic status, proficiency of English, household income, BMI category, dietary avoidances, and physical activity level. The table has 21 rows and 12 columns. Column headers include Age, Male, Country of birth, Region of residency, SEIFA, Proficiency of English, Equivalised income of household, BMI category, Dietary avoidances, and Physical activity level. Row labels include categories such as Major city, Inner regional area, Lowest 20 percent, Mainly speaks English at home, First decile, Underweight, All animal products, and Low. Each row provides percentages and mean values for all adolescent respondents, adolescent pulse consumers, and adolescent non-consumers. Notable trends include the majority of adolescents being born in Australia, residing in major cities, and speaking English at home. Most adolescents reported a healthy BMI and did not restrict their diet due to cultural, religious, or ethical reasons.

Navigate back to Table 1.
