# Supplementary material for: Pulse consumption of Australian adolescents: characteristics and consumption patterns in a national survey
Source: Br J Nutr. 2026 Jan 26;135(9):983–94. doi: 10.1017/S0007114526106308 (PMC13315545; doi:10.1017/S0007114526106308)
Supplement: Lanham et al. supplementary material [file S0007114526106308sup001.docx]

Demographical analysis

Nutritional analysis

Excluded from analysis due to small sample size

National Health Measures Survey (NHMS) (biochemical markers)

(n=11,000)

Adolescent (12-17 year old) NHMS participants only

(n=16)

Pulse consumption patterns analysis

Non-consumers

(n=943)

Pulse consumers

(n=64)

Included in demographical analysis (all adolescent participants)

(n=1,007)

Included in description of pulse consumption patterns

(n=64)

Pulse consumers

(n=49)

Non-consumers

(n=633)

Adolescent (12-17 year old) NNPAS participants only

(n=1,007)

Included in nutritional analysis

(n=682) including

Excluded participants: under- and over-reporters (n=152 and n=5, respectively), and participants missing information to be assessed for reporting accuracy (n=168))

National Nutrition and Physical Activity Survey (NNPAS) participants

(n=12,153)
